# To Remove Vulcanite

**Published:** 1900-02-15

**Authors:** A. E. H. Lister


### TO REMOVE VULCANITE.

To remove vulcanite from between the teeth; take a fine needle and mount it in a small handle or broach holder, sharpen it on two sides, and you have a useful tool for this purpose.—*A. E. H. Lister.*

